# A repetitive acidic region contributes to the extremely rapid degradation of the cell-context essential protein TRIM52

**DOI:** 10.1038/s41598-019-44359-0

**Published:** 2019-05-27

**Authors:** Kathrin Hacker, Stefan Benke, Benedikt Agerer, Sara Scinicariello, Valentina Budroni, Gijs A. Versteeg

**Affiliations:** 1 Department of Microbiology, Immunobiology, and Genetics, Max F. Perutz Laboratories (MFPL), University of Vienna, Vienna Biocenter (VBC), Dr. Bohr-Gasse 9, 1030 Vienna, Austria; 2 Present Address: CeMM Research Center for Molecular Medicine of the Austrian Academy of Sciences, Lazarettgasse 14 AKH BT25.3, 1090 Vienna, Austria

**Keywords:** Proteasome, Proteasome

## Abstract

Tripartite motif protein 52 (TRIM52) is a non-canonical TRIM family member harbouring the largest RING domain encoded in the human genome. In humans TRIM52 is conserved and has been under positive selection pressure, yet it has been lost in many non-primates. Competitive cell fitness assays demonstrated that *TRIM52* ablation reduces cellular fitness in multiple different cell types. To better understand how this cell-essential factor is controlled, we investigated how expression of this non-canonical protein is regulated. Here, we show that *TRIM52* mRNA is constitutively expressed from an intergenic region preceding the *TRIM52* gene. Yet, TRIM52 protein is rapidly turned-over by the proteasome with a 3.5-minute half-life, one of the shortest in the human proteome. Consistent with this extremely rapid degradation rate, all three TRIM52 domains were identified to contribute to its instability. Intriguingly, a repetitive acidic loop in the RING domain was identified as one of the main destabilizing regions, which was unexpected given the prevailing notion that these sequences are poor proteasome substrates. This work indicates that the effect of such repetitive acidic regions on proteasomal degradation depends on the protein context, and it identifies TRIM52 as an attractive model protein to study what these contextual properties are.

## Introduction

Steady-state eukaryotic protein levels are for an important part controlled by *de novo* synthesis on the one hand, and degradation by the proteasome on the other. Poly-ubiquitination plays a key role in targeting proteins to the proteasome for degradation^1^. In addition, efficient proteasomal degradation requires disordered regions in the target protein for initiating efficient feeding into the proteasome^2,3^. However, recent studies have indicated that unstructured protein regions with repetitive amino acid sequences are poor proteasome targets, providing insight into why disease-associated, toxic proteins like huntingtin may not be efficiently cleared and therefore accumulate in cells^4^.

The human genome encodes for approximately 600 proteins with a Really Interesting New Gene (RING) domain, a hallmark of many ubiquitin E3 ligase enzymes^5^. RING domains are zinc-fingers in which eight cysteine and histidine residues coordinate two zinc ions; these sites are connected in virtually all RING proteins through two similarly sized protein loops of usually five to twenty-five amino acids^5^. A few proteins harbour unique non-canonical RING domains that are substantially larger than all others, and/or have disproportionally sized loop regions.

Tripartite motif protein 52 (TRIM52) contains the largest known human RING domain^6^. Its extreme size stems from a substantially increased loop 2 region of 139 amino acids. Approximately a third of this extended loop 2 consists of acidic D/E residues, which predicts it to be unstructured at physiological pH^6^.

Phylogenetic analysis has revealed that the *TRIM52* gene arose in mammals only recently in evolution through partial gene duplication^6^. Subsequently, the *TRIM52* gene was lost or pseudogenised in many mammals. Yet, in primates the *TRIM52* gene has been maintained under positive selection pressure^6^. This had led to the notion that TRIM52 may play a biological role in immune-related processes or viral restriction, but had been thought unlikely to fulfil a significant cell-essential function^6,7^.

Unexpectedly, we recently found that *TRIM52* ablation is detrimental for cell cycle progression and thus proliferation in certain human cancer cell lines in cell culture and mouse xenograft models, without affecting cell viability^8^. Subsequently, similar observations have been reported by independent groups, positioning *TRIM52* as an important regulator of cell proliferation^9,10^. Epistasis experiments from our group, as well as independent studies indicate that a *TRIM52*-ablation phenotype requires a wild-type *TP53* allele (encoding the p53 protein)^8,9^. TRIM52 has been reported to ubiquitinate the p53-inhibitor PPM1A, thereby suppressing p53-dependent cell-cycle arrest^9^. In line with this observation, increased p53 activity and associated cell cycle inhibition has been put forward as a mechanism underlying the *TRIM52*-ablation phenotype^9^.

In line with a cell fitness-promoting physiological TRIM52 function, its expression has been found to be upregulated in several cancers^9,10^. Thus, while there is a strengthening notion that TRIM52 has cell essential proliferative functions –possibly explaining why it has been under positive selection pressure in humans–, and its expression is deregulated in cancers, how TRIM52 expression is regulated has remained unexplored.

Here, we report that *TRIM52* mRNA is expressed to readily-detectable, comparable medium-low levels in all tested cell lines, yet the protein is rapidly turned over by the proteasome. We identify three domains in the TRIM52 protein contributing to a three- to three-and-a-half-minute protein half-life, among the shortest known^11–15^. Unexpectedly, we find that instability is in part conferred by the acidic loop 2 region in the TRIM52 RING domain, which is similar to the acidic region in other proteins previously demonstrated to be poor proteasome substrates^4^.

## Results

### TRIM52 is ubiquitously expressed at low levels, yet is essential for cellular fitness

The overarching aim of this study was to investigate how *TRIM52* is itself regulated at the mRNA and protein levels. To learn general principles, rather than cell-line-specific regulation, multiple cell types with various tissue origins were interrogated.

**Figure 1:**
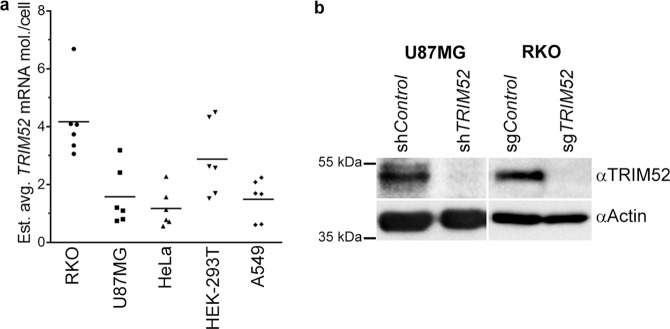
TRIM52 is ubiquitously expressed at low levels, yet essential for optimal cell fitness. (**a**) Following determination of cell numbers, total RNA isolated from five cell lines was analysed by RT-qPCR for *TRIM52* mRNA levels. In parallel *TRIM52* cDNA plasmid dilutions were analysed to generate a standard curve, from which absolute average *TRIM52* mRNA molecules per cell were estimated. Cellular qPCR detection ranged from ~16 cycles for *ACTB*, to ~28 cycles for *TRIM52*. Bars represent means; n = 6. (**b**) Western blot of U87MG glioblastoma cells expressing an shRNA targeting *TRIM52* or a non-targeting control shRNA (left panel), and RKO colon carcinoma cells expressing Cas9 and either an sgRNA targeting *TRIM52*, or a non-targeting control (right panel).

To this end, *TRIM52* mRNA levels were determined in five different cell lines from a known number of cells using RT-qPCR, relative to a standard dilution range of a *TRIM52* DNA plasmid with known numbers of molecules. This absolute quantification revealed that *TRIM52* mRNA is expressed at similar medium-low levels in all tested cell lines. Assuming a normal distribution, we estimate an average between approximately one and five *TRIM52* mRNA molecules per cell (Fig. 1a). However, based on quantification of multiple mRNAs from single-cell mRNA-seq studies, the population distribution may range from some cells having no *TRIM52* mRNA, to several hundred copies per cell^16^.

In contrast to readily detectable *TRIM52* mRNA levels, Western blot (WB) analyses required long exposure times, which suggested that the TRIM52 protein is present at low steady-state concentrations (Fig. 1b; left lanes). Detection of exogenously expressed OLLAS- or MYC-tagged TRIM52 was superior using the A-4 αTRIM52 mAb, compared to the considered sensitive OLLAS and MYC mAbs (Fig. S1a)^17,18^, indicating that low levels of detected endogenous TRIM52 (Fig. 1b) did not result from antibody insensitivity. Moreover, *TRIM52* ablation by two independent methods (RNA interference or CRISPR-mediated knock out) abrogated TRIM52 detection by WB in U87MG and RKO cells (Fig. 1b; right lanes), attesting to specificity of the mAb.

Next, the effects of *TRIM52* ablation on the cell were investigated. *TRIM52* ablation significantly decreased cellular fitness of both cell types in a competition assay (Fig. S1b). This establishes the unrelated neural-origin U87MG and intestinal-origin RKO cell lines as good cell models in which TRIM52 plays a biological function, for studying *TRIM52* mRNA and protein regulation.

### *TRIM52* mRNA is expressed from a gene-adjacent intergenic region

**Figure 2:**
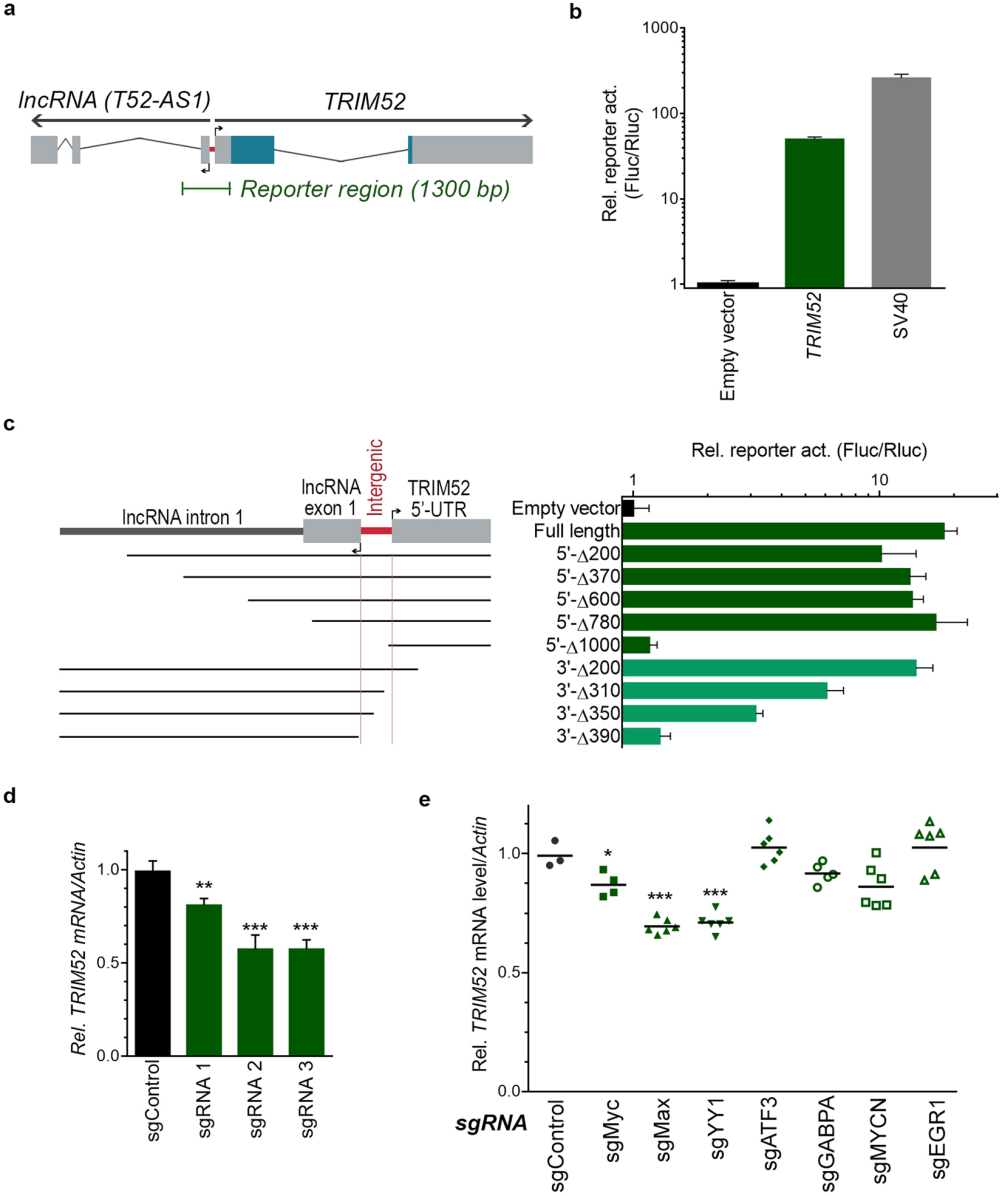
*TRIM52* mRNA is expressed at moderate-low levels in different cell types from a gene-adjacent 93 bp intergenic region. (**a**) Schematic overview of the *TRIM52*, and the adjacent sequence-unrelated *TRIM52-AS1* lncRNA loci. The 93 bp intergenic region is indicated in red. Wide grey bars are exons, thin bars introns. Blue colour indicates protein coding reading frame. Green indicates the 1300 bp fragment analysed by reporter assays. (**b**) A firefly luciferase reporter containing the 1300 bp region upstream of the *TRIM52* start codon was co-transfected with a constitutively expressed renilla luciferase plasmid in HEK-293T cells. Subsequently, dual luciferase measurements were performed and F_luc_/R_luc_ ratios plotted. Data represent means and s.d. n = 3. (**c**) Dual luciferase assays performed with 5′- and 3′- deletion constructs of the *TRIM52* transcriptional reporter in U87MG cells. Data represent means and s.d. n = 3. (**d**) The identified 93 bp *TRIM52* transcriptional region was targeted in RKO cells using CRISPR with three independent sgRNAs. Relative *TRIM52* mRNA levels were determined by RT-qPCR. Data represent means and s.d.; n = 3. **p = 0.01, ***p = 0.001. (**e**) The indicated transcription factors were targeted in RKO cells using CRISPR with two independent sgRNAs/gene. Relative *TRIM52* mRNA levels were determined by RT-qPCR. Data represent means of combined data from two sgRNAs/gene; n = 6. *p = 0.03, ***p = 0.0001.

We hypothesized that *TRIM52* is constitutively transcribed to achieve the similar *TRIM52* mRNA levels found in all tested cell lines (Fig. 1a). Analysis of the human *TRIM52* locus using the ECR browser platform^19^ revealed transcriptional start sites of *TRIM52* and the sequence-unrelated *TRIM52-AS1* lncRNA on the opposite strand, separated only by a 93 bp intergenic region (Fig. 2a; indicated in red). This intergenic region is conserved in primates in which *TRIM52* is maintained under positive selection pressure, but not in non-maintaining species (not shown)^6^. From this unusually tight gene-spacing it remained unclear which genomic region drives *TRIM52* transcription.

To identify the region driving *TRIM52* mRNA expression, the 1300 bp region upstream of the *TRIM52* start codon (Fig. 2b) was cloned in a luciferase transcriptional reporter. Transfection in HEK-293T cells reproducibly activated expression of the reporter 50–100 fold over the empty vector (Fig. 2b), indicating that this region harbours constitutive transcriptional activity. We then progressively deleted segments of this 1300 bp *cis-*regulatory region to map the minimal sequence able to drive transcription of the reporter, in both HEK-293T (Fig. S2a) and U87MG cells (Fig. 2c). In both cell lines, removal of the 5′ most 780 bp or the 3′ most 200 bp from the *cis*-regulatory region, did not change transcriptional output compared to the full-length reporter (Figs 2c and S2a). In contrast, transcriptional output progressively declined in mutants that deleted parts of the 93 bp intergenic region (Figs 2c and S2a). Together, these results indicate that the intergenic region directly adjacent of the *TRIM52* transcriptional start site (TSS) is sufficient to drive transcription.

To test whether the identified intergenic region indeed is also required for *TRIM52* mRNA expression, the endogenous intergenic region was targeted in RKO cells using CRISPR with three independent sgRNAs (Fig. 2d). All three sgRNAs significantly decreased *TRIM52* mRNA expression by RT-qPCR by 20% for sgRNA1, and 45% for sgRNAs 2 and 3 (Fig. 2d). Taken together, from these data we conclude that an important part of constitutive *TRIM52* transcription is driven from the intergenic region directly upstream of the TSS. The fact that reporter activity is progressively lost with 3′ deletions, and sgRNA targeting (which in most cases deletes only one or a few bp) only partially ablated *TRIM52* mRNA expression, suggests that multiple transcription factor binding sites may be present in the intergenic region, which in concert are responsible for constitutive *TRIM52* expression.

To identify transcription factors (TFs) driving *TRIM52* transcription, we analysed publicly available ChIP-seq data in the Genome Browser with ReMap^20^ to identify TFs and transcriptional co-factors which have been experimentally identified to bind to the *TRIM52* intergenic region across many different cell types. Thirty-two different TFs and co-factors were identified twice or more across different experiments at the *TRIM52* intergenic region (Fig. S2b). The identification of such a wide range of factors suggested that various TFs could contribute to *TRIM52* transcription, possibly in partially redundant capacities. From these factors we selected seven TFs which were identified with varying frequency, ranging from nearly 30 times for cMYC (out of 196 measured events) to just over the lower cut-off limit for EGR1, to assess whether their ablation directly or indirectly altered *TRIM52* mRNA levels (Fig. S2b; green bars). To this end, each TF was targeted in RKO cells with two independent sgRNAs using CRISPR, after which *TRIM52* mRNA levels were determined by RT-qPCR.

Targeting of the three most-frequently identified TFs (cMYC, MAX, YY1; Fig. S2b) significantly reduced *TRIM52* mRNA levels by 10% (cMYC) to 35% (MAX, YY1; Fig. 2e). Together, the reporter analyses, ChIP-seq data and knock-out experiments suggest that *TRIM52* mRNA expression is driven by multiple TFs, including cMYC, MAX and YYI, from the intergenic region located between the *TRIM52* and *TRIM52-AS1* lncRNA genes.

### TRIM52 is upregulated during complex cell stress

Since *TRIM52* has been under positive selection pressure in primates (often a signature of immune- or stress-response genes)^6,7^, and many other TRIM proteins have been reported to be induced by cytokines and during infections^21,22^, we asked whether TRIM52 expression is increased under these stimulatory conditions.

**Figure 3:**
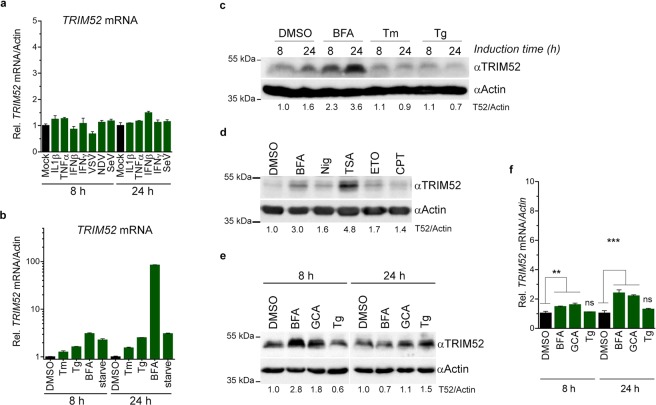
TRIM52 is upregulated during complex cell stress induced by Golgi toxins. (**a**) U87MG cells were treated/infected with the indicated cytokines/viruses. After 8 and 24 h, *TRIM52* mRNA levels were determined by RT-qPCR. (**b**) U87MG cells were treated for 8 or 24 h with tunicamycin (Tm), thapsigargin (Tg), or brefeldin A (BFA), after which *TRIM52* mRNA levels were determined by RT-qPCR. (**c**) Parallel samples from (b) were analysed by WB for TRIM52. Numbers under the lanes indicate TRIM52 WB densitometry quantification relative to actin. (**d**) U87MG cells were treated with the indicated compounds reported to induce Golgi stress, after which cell lysates were harvested at 24 h and analysed by WB. **(e,f)** RKO cells we treated with the Golgi stress inducers BFA or golgicide A (GCA) for 8 or 24 h, after which **(e)** TRIM52 protein levels by WB, or (**f**) mRNA levels of *TRIM52* by RT-qPCR were determined. Numbers underneath the WB lanes indicate TRIM52 WB densitometry quantification relative to actin. RT-qPCR data represent means and s.d.; n = 3. **p = 0.01, ***p = 0.001.

U87MG cells were treated for 8 or 24 h with the indicated cytokines, or infected with Sendai virus (SeV), vesicular stomatitis virus expressing GFP (VSV), or Newcastle disease virus expressing GFP (NDV). However, none of these stimuli induced *TRIM52* mRNA levels (Fig. 3a), despite all cytokines successfully inducing known response genes (Fig. S3a), and the viruses achieving productive infection as determined by SeV defective-interfering RNA expression (Fig. S3a; right panel), and GFP expression in >95% of VSV- and NDV-infected cells (not shown).

To identify conditions during which *TRIM52* could be induced, we interrogated published micro-array and mRNA-seq data sets, and identified significant and robust *TRIM52* mRNA upregulation in U87MG cells during a late time point (16 h) of infection with the human high-containment pathogen Venezuelan encephalitis virus^23^. Interestingly, *TRIM52* expression was not significantly changed at earlier time points (4–8 h) when cytokines and immune-related response genes were strongly induced^23^. Based on this observation we hypothesised that *TRIM52* could be induced during the complex cellular stress caused by such infection. In particular, many acute viral infections overload the cellular export system with viral proteins during late stages of infection, causing ER and Golgi stress^24^.

Therefore, we next investigated whether chemical induction of the ER Unfolded Protein Response (UPR) by tunicamycin (Tm) or thapsigargin (Tg), or treatment with brefeldin A (BFA; a Golgi toxin inducing both the UPR and Golgi stress) would increase *TRIM52* mRNA expression. In addition, cells were nutrient-starved in Earle’s balanced salt solution (EBSS), as an alternative inducer of ER stress and autophagy^25^. All stimuli induced a robust UPR at 8 and 24 h as evidenced by non-canonical cytoplasmic *XBP1* mRNA splicing by the IRE1-branch of the UPR (Fig. S3b)^26,27^, and strong upregulation of the known UPR-genes *HSPA5* and *GADD34* (Fig. S3c)^28^. However, only the Golgi toxin BFA substantially induced *TRIM52* mRNA levels (90-fold; Fig. 3b) at 24 h post-induction, but not at 8 h post-treatment. TRIM52 protein was upregulated by BFA at 8 h (2.3 fold), which further increased by 24 h post-treatment (3.6-fold; Fig. 3c). This indicates that robust UPR induction *per se* does not induce TRIM52. Instead, it suggested that TRIM52 can be induced at the protein levels in response to Golgi stress, and/or resulting downstream responses at 8 h, which may be enhanced at later time points by increased mRNA expression.

A cellular Golgi stress response distinct from the ER-specific UPR has been previously reported^29–31^. This response is characterized by a relatively late transcriptional onset after induction (18–24 h)^30^, which is in line with the observed *TRIM52* response (Fig. 3b,c). In addition to BFA, the ionophore nigericin (Nig), the HDAC inhibitor trichostatin A (TSA), and topoisomerase inhibitors etoposide (ETO) and campothecin (CPT) have been shown to induce this characteristic Golgi stress response^31^. To investigate if these Golgi stress inducers would also increase TRIM52 protein levels, U87MG cells were stimulated for 24 h. Indeed, stimulation with all five inducers increased TRIM52 levels, albeit to varying degrees (Fig. 3d).

Lastly, to determine whether TRIM52 induction by Golgi toxins is also elicited in other cell lines, RKO cells were treated with Golgi toxins BFA and golgicide A (GCA; structurally different from BFA, yet targeting the same pathway^32^), and the UPR inducer Tg. All three stimuli significantly induced the UPR evidenced by the transcriptional induction of *HSPA5* at 8 and 24 h (Fig. S3d). Attesting to a Golgi-toxin specific effect, TRIM52 protein was only increased in RKO cells by BFA and GCA to a similar extent as in U87MG cells, yet unexpectedly only at the earlier 8 h time point (Fig. 3e). At the mRNA level, *TRIM52* was almost not changed in RKO cells at both 8 h and 24 h (<3 fold; Fig. 3f), which differs from the strong 24 h increase in U87MG cells.

Thus, the ability of Golgi toxins to increase TRIM52 protein levels at 8 h without prominent changes in mRNA levels is shared between both cell lines, suggesting that this may be determined by post-translational mechanisms at the protein level. Yet, increased TRIM52 protein at 24 h was only found in U87MG cells co-occurring with a strong rise in mRNA concentrations, indicating that this increase in transcripts could contribute to maintaining increased TRIM52 protein levels at this time point.

### TRIM52 protein is rapidly degraded by the proteasome

**Figure 4:**
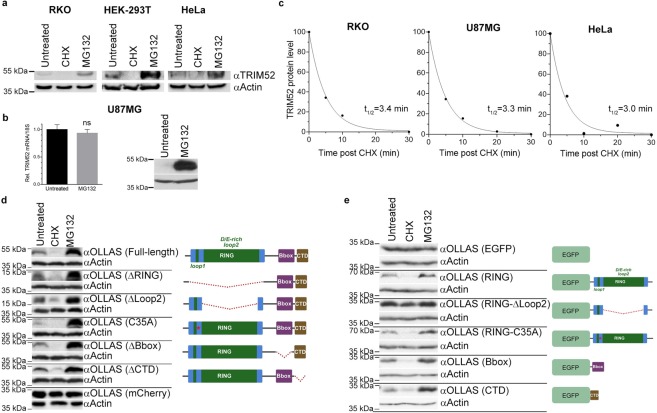
TRIM52 protein is present at low steady-state levels as a result of rapid proteasomal turn-over. (**a**) The indicated cell lines were treated with the translation inhibitor cycloheximide (CHX) or proteasome inhibitor MG132 for 3 h; whole-cell lysates were analysed by WB. (**b**) U87MG cells were treated with MG132 for 6 h, after which *TRIM52* mRNA levels were determined by RT-qPCR, and protein levels by WB. Data represent means and s.d.; n = 3. (**c**) TRIM52 protein levels were determined at the indicated time points by WB following CHX chase. WB bands were quantified, normalized to actin levels, and values plotted. A single-exponential decay curve was fit and used to calculate half-lives. (**d**) HEK-293T cells were transfected with plasmids encoding OLLAS-tagged full-length TRIM52, or one of the indicated deletion- or RING-disrupting point-mutants. Cells were treated with CHX or MG132 for 3 h, after which whole cell lysates were analysed by WB. (**e**) HEK-293T cells were transfected with plasmids encoding OLLAS-tagged EGFP, or EGFP-fusions with the indicated TRIM52 domains. Cells were treated with CHX or MG132 for 3 h, and EGFP levels determined by WB.

Despite *TRIM52* mRNA being readily detectable by RT-qPCR (Fig. 1a), steady-state TRIM52 protein levels determined by WB were estimated to be low in the same cell lines (Fig. 1b). We hypothesised that TRIM52 is readily translated, yet rapidly turned-over. Indeed, upon treatment of RKO, HEK-293T, and HeLa cells for 3 h with the translation inhibitor cycloheximide (CHX), TRIM52 protein was lost (Fig. 4a; middle lanes). On the other hand, inhibition of proteasomal activity with MG132 (Fig. 4a; right lanes), or the two unrelated proteasome inhibitors epoxomicin and bortezomib (Fig. S4a), increased TRIM52 levels, indicating that degradation by the proteasome contributes to its low steady-state protein levels. Likewise, proteasome and translation inhibition similarly affected TRIM52 protein levels in U87MG cells (Fig. 4b; right panel). In contrast, *TRIM52* mRNA remained unaffected by the same MG132 treatment (Fig. 4b; left panel), demonstrating that the effect of the inhibitor occurs at the protein, and not the mRNA level. A subsequent CHX chase analysis up to 30 minutes showed a rapid single-exponential decay with calculated TRIM52 protein half-lives measured in all three cell lines between 3 and 3.5 minutes (Fig. 4c). Together, these results show that TRIM52 is readily produced in these cell lines, yet is rapidly degraded by the proteasome.

To identify the protein regions which contribute to the extreme TRIM52 protein turn-over rate, we analysed a number of domain deletions (Fig. 4d). In addition to deletion of full domains, we also tested the effect of deleting the D/E-rich loop 2 within the RING domain, and of introducing a C-to-A mutation expected to ablate ubiquitin E3 ligase activity^33^. These mutants were expressed in HEK-293T cells at levels comparable to endogenous TRIM52.

Expression of full-length N-terminally OLLAS-tagged TRIM52 had degradation characteristics similar to endogenous TRIM52: CHX treatment resulted in rapid loss of detectable protein, whereas proteasome inhibition stabilised it (Fig. 4d; top). Similarly, each of the single-domain mutants displayed the same stability behaviour, whereas the steady-state level of a known stable protein (mCherry; Fig. 4d, bottom) remained unaffected by both treatments. Intriguingly, these results indicate that it is not an individual domain of TRIM52 that contributes to degradation by the proteasome.

Lastly, in order to identify the protein regions contributing to TRIM52 instability, individual TRIM52 domains were fused to the C-terminus of the stable EGFP protein (Fig. 4e). As expected, non-fused EGFP levels remained stable during CHX treatment and proteasome inhibition (Fig. 4e; top). In contrast, fusion of any of the three individual conserved TRIM52 domains (RING, Bbox, or CTD) to EGFP, rendered it unstable upon translation inhibition, while stabilising it by MG132 (Fig. 4e), thus phenocopying the behaviour of full-length TRIM52. A point-mutation in the RING domain disrupting putative E3 ligase activity was also unstable, indicating that TRIM52 E3 activity is likely not required for instability conferred by the RING domain. Unexpectedly, deletion of the repetitive D/E-rich loop 2 substantially stabilized the EGFP-fusion, indicating that this is the predominant destabilising region in the RING domain.

Together, these data establish that TRIM52 protein is present in various cell types at low steady-state levels resulting from rapid proteasomal turn-over mediated by all three identified TRIM52 domains. The non-canonical RING domain is one of the destabilizing regions, which is independent of its E3 ligase function, yet unexpectedly conferred by the repetitive loop 2. This work establishes TRIM52 as an interesting model protein to study protein degradation concepts, and provides a starting point to better understand how intrinsic and extrinsic factors contribute to this.

## Discussion

Several independent studies have recently identified TRIM52 as an essential factor in various cancer cell lines, whose expression is deregulated in primary cancer samples^8–10^. In this study we characterized how TRIM52 itself is regulated. Our data show that while constitutively transcribed, steady-state TRIM52 protein levels are low as a result of very rapid turn-over by the proteasome. Stability assays revealed that all three domains in the TRIM52 protein contribute to an extremely fast three- to three-and-a-half-minute protein half-life, among the shortest known^11–15^. Unexpectedly, this instability is in part mediated by a repetitive acid loop 2 in the non-canonical RING domain, a sequence which would be predicted to be a poor proteasome substrate based on current knowledge^4^.

Our data show that *TRIM52* mRNA is expressed to similar medium-low levels in different cell types, in part from the small 93 bp intergenic region preceding the *TRIM52* gene. Analysis of publicly available ChIP-seq data from many independent experiments in various different cell types indicated that this region is found to contain many different transcription factors. This could indicate that many of these identified factors contribute redundantly to the baseline *TRIM52* mRNA expression observed in all tested cell lines. In line with this notion of functional redundancy, cMYC, MAX and YY1 targeting by CRISPR each significantly, yet to a limited degree, decreased cellular *TRIM52* mRNA content. Moreover, several general chromatin modifiers and transcriptional repressors were found to interact with the *TRIM52* transcriptional region. This opens up the possibility that *TRIM52* mRNA levels are in addition controlled by transcriptional repression.

Furthermore, we report that complex cell stress –such as caused by Golgi toxins- increases TRIM52 levels, albeit to various degrees depending on the cell line. On the one hand, we show that induction of the fast and transcriptionally well-defined UPR does not affect cellular *TRIM52* mRNA concentrations. In contrast, the Golgi stress response is less well-defined; its reported transcriptional targets have a higher degree of heterogeneity, and it occurs mainly at later time points after toxin exposure^30,31^. This relatively late response thus likely represents a combination of genes involved in Golgi maintenance and structure, as well as unrelated general stress response genes which control the cellular decision making between recovery and resolution of stress on the one hand, and apoptosis on the other.

Thus, while our data indicate some specificity to the treatments which upregulate *TRIM52* mRNA in U87MG cells, we cannot definitively conclude whether this results from direct effects on Golgi structure, or is instead driven by the general complex cell stress associated with these toxins. In addition, we found that some cell lines have a strong late *TRIM52* mRNA response (U87MG), whereas others do not (RKO). Nevertheless, the early increase in TRIM52 protein is similar in all circumstances, which raises the question whether this results from increased protein stability. However, double-treatments with Golgi toxins and CHX to address whether protein synthesis is required for the increase in TRIM52 protein levels have thus far been unsuccessful stemming from the high combined toxicity of these drugs. Likewise, experiments with the Pol II inhibitor actinomycin D to address whether the late *TRIM52* mRNA increase in U87MG cells depends on *de novo* mRNA synthesis have been hampered by the high cellular toxicity of combined treatment with two toxins.

Finally, we show that TRIM52 protein steady-state levels are low as a result from rapid turn-over by the proteasome. Our measurements put TRIM52 protein half-life at 3–3.5 minutes. Proteome-wide half-life determination in yeast and human cells have identified only less than five proteins with comparably short half-lives^14,34^. For example, TRIM52 is substantially faster degraded than cMYC (half-life 10–30 min.^11,12,35^) and HIF1α (half-life 5–10 minutes^13^) which are generally considered to be among the fastest turned-over cellular proteins. This identifies TRIM52 as a protein with extreme properties both in terms of protein domain organisation, and protein regulation. Consistent with this fast degradation rate, all three TRIM52 domains were shown to contribute to its instability.

The repetitive D/E-rich loop 2 in the extended RING domain was identified as a main destabilizing region, which was unexpected given the prevailing notion that these sequences are poor degradation substrates as they are inefficiently fed into the proteasome^4^. In particular, the E2 conjugase CDC34 has a disordered C-terminal tail with similar sequence composition as loop 2 in the TRIM52 RING, yet a fusion with this region did not destabilize a reporter protein^4^. This indicates that certain intrinsic protein properties can determine proteasomal degradation in a protein-context dependent manner.

Disordered protein regions are often associated with rapid proteasomal turnover, yet there are examples which are stable^36,37^. Repetitive sequences as found in the TRIM52 loop2 and CDC34 tail are comparable examples, yet how their differential cellular fate is mechanistically determined remains for an important part unknown. Together, our results position TRIM52 as an interesting model protein to study how highly turned-over proteins are degraded, and what the extrinsic and protein-intrinsic factors are that contribute to this, and ultimately better understand the concepts of how they determine protein degradation in some proteins but not others.

## Materials

### Reagents

The following antibodies were used for Western blot analyses. Actin (Abcam, ab49900); TRIM52 (Santa Cruz Biotechnology, sc-398954); OLLAS (Novus Biologicals, NBP1-06713); anti-mouse IgG-HRP (Cell Signaling Technology); anti-rat-IgG-HRP (Abcam, ab97057). Sendai virus Cantell (ATCC) and NDV-GFP La Sota were grown on 10-day old embryonated chicken eggs. VSV-GFP Indiana was grown on Vero cells. SeV was tittered by IFNβ-reporter assay in HEK-293T cells as previously described. NDV-GFP and VSV-GFP were both tittered by GFP-based TCID50 assay in HEK-293T.

### Cells culture and treatments

All cells were cultured in high glucose Dulbecco’s Modified Eagle’s Medium (DMEM; Sigma-Aldrich, D6429) containing 10% foetal bovine serum (FCS; Sigma-Aldrich, F7524) and 1% Penicillin-Streptomycin (Sigma-Aldrich, P4333) and at 37 °C and 5% CO_2_ in a humidified incubator. Cells were treated with the following reagents for indicated time points: 200 µg/ml cycloheximide (Fluka, 01810); 100 ng/ml brefeldin A (Sigma Aldrich B5936); 10 µM MG-132 (Sigma; M7449); 10 µM epoxomicin (Gentaur; 607-A2606); 10 µM bortezomib (Selleck Chemicals; PS-341); 2 µg/ml (U87MG) or 100 ng/ml (RKO) doxycycline (Sigma-Aldrich; D9891); 10 ng/ml human TNF-α (Peprotech; 300-01A); 1 µg/ml tunicamycin (Sigma, T7765); 1 µg/ml thapsigargin (Sigma, T9033); 10 ng/ml human IL-1β (R&D systems, 201-LB-025); 50 IU/ml human IFNβ (PBL, 11420-1); 10 ng/ml IFNγ (PBL, -11500-2).

### Western blot analysis and densitometry analysis

Cells were lysed using a disruption buffer containing 2.1 M Urea, 667 mM β-mercaptoethanol and 1.4% SDS. Cell lysates were boiled at 98 °C for 10 min and DNA was sheared by passing lysates through an 30 G needle. Proteins were separated via SDS-PAGE using 10% polyacrylamide gels and transferred onto nitrocellulose membranes, and blocked for 2 h with TBS supplemented with 5% BSA. Horseradish peroxidase conjugated antibodies were used and bands were visualized using ECL buffer (100 mM Tris (pH 8.5), 0.07% Coumaric acid (Sigma-Aldrich, C9008), 0.44% Luminol (Sigma-Aldrich, A8511) on a ChemiDoc Touch Imaging System (BioRad). Relative protein levels were quantified using Image Lab (BioRad). Background was acquired and subtracted from density volumes for TRIM52 and actin. Subsequently, TRIM52 densities were normalized to actin, and subsequently to treatment controls.

### Protein half-life determination

Cells were treated with cycloheximide as described above. At indicated time points, whole cell lysates were prepared, analysed by WB, and quantified and normalized to actin levels as indicated above, and previously described^38^. Single exponential decay curves were determined using Graphpad Prism, from which protein half-lives were calculated.

### Competitive cellular fitness assay

Competitive cell fitness assays were performed as described previously^39^. In brief, 70% U87MG cells containing dox-inducible *TRIM52*-targeting or non-targeting shRNAs, or RKO cells harbouring dox-inducible Cas9 and expression plasmids for expression of *TRIM52*-targeting or non-targeting sgRNAs were mixed with 30% WT cells. Targeting sequences are provided in the supplementary information. Media were supplemented with dox (U87MG: 2 μg/ml; RKO: 100 ng/ml). The following day, the percentage fluorescent cells was measured by flow cytometry. Cells were split every two days in the presence of dox in a 1:2 ratio (U87MG) or 1:5 ratio (RKO), respectively, and fluorescence signal was measured by flow cytometry on these same days. Data from each measurement were normalized to the percentage fluorescence-positive cells from each individual cell pool on the day of induction with dox.

### Transcriptional dual-luciferase reporter assay

HEK-293T cells in M24 well clusters were transfected with 150 ng Fluc reporter and 50 ng pRL-TK (Rluc; Promega). U87MG cells in a M24 well cluster were transfected with 350 ng Fluc reporter and 150 ng pRL-TK. Forty-eight hrs after transfection, cells were lysed with Passive Lysis Buffer (Promega, E194A) and Firefly- and Renilla luciferase activities measured in a dual-luciferase assay using a Synergy H1 plate reader (BioTek). Substrates were prepared as previously reported^40^.

### Statistical analyses

GraphPad Prism was used to calculate statistics using two-tailed student’s t-test. A p-value < 0.05 was considered statistically significant. All values are represented as mean ± s.d.

### XBP1 splicing assay

After RNA isolation, and cDNA preparation, XBP1 was PCR amplified with the following primers^27^: Fwd-5′-aaacagagtagcagctcagactgc-3′ and Rev-5′-tccttctgggtagacctctggga-3′ using this PCR program: 95 °C - 5 min, (95 °C - 20 s, 60 °C - 30 s, 72 °C - 30 s) for 35 cycles, 72 °C-10 min). PCR amplicons were digested with PstI (NEB R3140) for 1 h at 37 °C, and subsequently analysed on a 3% agarose gel.

## Supplementary information

Supplementary Information

## Data Availability

All data generated or analysed during this study are included in this published article (and its Supplementary Information files).
