# Supplementary material for: A repetitive acidic region contributes to the extremely rapid degradation of the cell-context essential protein TRIM52
Source: Sci Rep. 2019 May 27;9:7901. doi: 10.1038/s41598-019-44359-0 (PMC6536530; doi:10.1038/s41598-019-44359-0)
Supplement: Supplementary file 1 — Supplementary Information [file 41598_2019_44359_MOESM1_ESM.pdf]

A repetitive acidic region contributes to the extremely rapid degradation of the cell-context essential protein TRIM52

Kathrin Hacker[1], Stefan Benke[1], Benedikt Agerer[1,2], Sara Scinicariello[1], Valentina Budroni[1], and Gijs A. Versteeg[1].

Supplementary Materials and Methods

Transfections

Transfection mixes were made containing DNA and Polyethylenimine (PEI; Polysciences, 23966) in a ratio of 3:1 (µg PEI/µg DNA) and serum-free DMEM. After incubation for 25 min at room temperature the mixes were added dropwise to the cells with full growth medium.

Identification of *TRIM52* intergenic region, TSS, and analysis of public ChIP-seq data

The ECR browser (https://ecrbrowser.dcode.org/)[5] was used to identify conserved mRNA encoding regions for the *TRIM52* and *TRIM52-AS1* lncRNA genes. In addition, the non-transcribed intergenic region was identified as the region to which no known transcripts were matched. For this analysis Homo sapiens, Bos taurus, Macaca mulatta, and Pan troglodytes were included, whereas Canis familiaris and all other species lacking a *TRIM52* gene were excluded. Subsequently, the *TRIM52* intergenic region with adjacent sequences was located in the UCSC Genome browser (https://genome-euro.ucsc.edu/; Dec. 2013 (GRCh38/hg38) Assembly). Public Encode and ReMap sub-tracks (hg38 assembly) were loaded, transcriptional regulators exported, regulators identified less than twice, or only in a single cell line were removed, and the identification frequency calculated for the remaining factors.

RNA isolation, cDNA synthesis, and RT-qPCR

RNA isolation was performed with TRIzol reagent (Thermo Fisher Scientific, 15596) following the manufacturer's protocol. Isolated RNA was treated with 3 units TURBO DNase (Thermo Fisher Scientific, AM2238) for 30 min at 37 °C, re-precipitated, and heat-inactivated at 75 °C

for 10 min Reverse transcription was performed using RevertAid Reverse Transcriptase (ThermoFisher, EP0441) and random primers (ThermoFisher, 48190011). qPCR was carried out in PCR buffer (10 mM Tris (pH 8.5), 50 mM KCl, 0.15% Triton X-100, 2 mM MgCl, 200 μM dNTPs (Promega U1515), 200 mM Trehalose (Sigma, T9531), 2.5% formamide (Sigma, 47670), 0.005% SYBR Green (Fisher, 10207252), 50 u/ml Taq-polymerase (Promega, M7848) in M384 plates on a Roche LightCycler 480. Cycling conditions: 95 °C - 5 min, (95 °C - 15 s, 56 °C - 15 s, 72 °C - 20 s) for 55 cycles. Subsequently, a melting curve was determined to ensure single amplicons in each reaction.

Table – qPCR primers

Species	Target	Direction	Sequence (5'-3')
Human	*ACTB*	Fwd	aggcaccagggcgtgat
Human	*ACTB*	Rev	gcccacataggaatccttctgac
Human	*18S rRNA*	Fwd	gtaacccgttgaaccccatt
Human	*18S rRNA*	Rev	ccatccaatcggtagtagcg
Human	*HSPA5*	Fwd	gtactgcttgatgtatgtcc
Human	*HSPA5*	Rev	accttcatagaccttgattg
Human	*GADD34*	Fwd	agacttctgcttccacac
Human	*GADD34*	Rev	cttccttatcctcactatcc
Human	*ISG56*	Fwd	ttgatgacgatgaaatgcctga
Human	*ISG56*	Rev	caggtcaccagactcctcac
Human	*ICAM1*	Fwd	gaaccagagccaggagacac
Human	*ICAM1*	Rev	cttcgtcagaatcacgttgg
Human	*IL1B*	Fwd	cgaccaccactacagcaagg
Human	*IL1B*	Rev	tcaaagatgaagggaaagaagg
Human	*SeV-DI*	Fwd	tccaagactatctttatctatgtcca
Human	*SeV-DI*	Rev	gcttcaaacttctggtcagg
Human	*TRIM52*	Fwd	ggtgcaggagtaccaggaaataa
Human	*TRIM52*	Rev	ataggccttgctgtgaatgct

Absolute *TRIM52* mRNA quantification

For absolute quantification of *TRIM52* mRNA content, qPCR on RT samples from a quantified number of cells (Countess, Invitrogen) was performed in parallel to a standard dilution series of a dsDNA expression plasmid harbouring a full-length *TRIM52* cDNA. For the standard dilution range, ten-fold dilutions were generated such that each qPCR reaction

per dilution received a dsDNA mass ranging from 10 ng to 1 fg. Using http://www.molbiotools.com/dnacalculator.html this was determined to be the equivalent of 1.55 x 10^9 molecules/ qPCR reaction for the 10 ng samples, to 155 molecules/ qPCR reaction for the 1 fg samples. A trend line was calculated to correlate detection cycle with absolute DNA molecules; the correlation coefficient of the dilution range in the qPCR analysis was R^2=0.999. For estimating the number of *TRIM52* mRNA molecules per cell, the relative quantification levels in RT reactions were increased by a factor of two to correct for starting with a single-stranded DNA template in the RT reactions, relative to the dsDNA template in the standard dilutions. Using the determined trend line formula, the absolute number of *TRIM52* molecules in each 75 ng of total RNA qPCR input was calculated. Finally, from the mass of total RNA isolated from a counted number of cells from which this was isolated, the average number of *TRIM52* mRNA molecules per cell was calculated.

Transduction and *TRIM52* targeting

Lentivirus-like particles (VLP) were produced by seeding HEK-293T cells at ~20% confluency into M6 clusters. The day after, cells were transfected with 500 ng psPAX2-GagPol plasmid, 500 ng mini genome and 100 ng pCMV2-VSV-G plasmid. Forty-eight hrs after transfection, recipient cells were seeded into M6 clusters. The subsequent day, VLP-containing supernatants were harvested, filtered through a 0.45 µM filter and diluted in growth media containing 8 µg/ml polybrene (Millipore, TR-1003-G). Media from recipient cells were replaced with VLP-containing media. Three days after transduction, cells were selected using 3 µg/ml puromycin (Fisher, 10296974) for at least three days and kept under puromycin selection. shRNA-mediated knock-down was achieved by VLP delivery of a doxycycline (dox)-inducible vector containing *TRIM52*- or Renilla luciferase (non-targeting) shRNA coding-sequences in the artificial 3'-UTR of EGFP as previously described[1,2]. shRNA target sequences are specified in the table below. To induce shRNA expression, cells were treated with 2 µg/ml dox (Sigma-Aldrich, D9891) for the duration of the assay. For CRISPR-mediated gene targeting sgRNA sequences were cloned in the BsmBI sites downstream of the U6

promoter as described[3]. Target sequences for sgRNAs are specified in the table below. RKO cells harbouring a dox-inducible Cas9 vector were transduced with VLPs for U6-promoter driven expression of sgRNAs[4]. To induce Cas9 expression, cells were treated with 100 ng/ml dox (Sigma-Aldrich; D9891). Subsequently, cells were analysed at indicated time points in competitive cell fitness assays, or three days post-induction lysates were prepared for WB.

Table - Target sequences shRNAs and sgRNAs

**Type**	**Target**	**Target sequence (5'-3')**
shRNA	*Renilla luciferase (control)*	taggaattataatgcttatcta
shRNA	*TRIM52*	atacgatgaggacgaagatgaa
sgRNA	*ATF3.1*	tgtcagcgacagacccctcg
sgRNA	*ATF3.2*	aaagtgccgaaacaagaaga
sgRNA	*EGR1.1*	gaaaatgtcagtgttcggcg
sgRNA	*EGR1.2*	ggacaactaccctaagctgg
sgRNA	*GABPA.1*	gagtgtggtgaggtctatat
sgRNA	*GABPA.2*	ctccagagaatttctccccg
sgRNA	*MAX.1*	atgcactggaacgaaaacgt
sgRNA	*MAX.2*	aatatatccagtatatgcga
sgRNA	*MYC.1*	cttcggggagacaacgacgg
sgRNA	*MYC.2*	ctatgacctcgactacgact
sgRNA	*MYCN.1*	gttcttgggacgcacagtga
sgRNA	*MYCN.2*	tgcatcctcactctccacgt
sgRNA	*TRIM52*	gagggtgtgtgacccagctg
sgRNA	*YY1.1*	actgcctgctatgccccctg
sgRNA	*YY1.2*	caaccccaatctcaatccgg
sgRNA	*TRIM52 transcriptional region.1*	tggtgtgccgggggccgcgc
sgRNA	*TRIM52 transcriptional region.2*	ggcggaggaccacgtctcta
sgRNA	*TRIM52 transcriptional region.3*	atagagacgtggtcctccgc

Generation of *TRIM52* transcriptional reporter constructs

The *TRIM52* transcriptional reporter region was amplified from HEK-293T cDNA by PCR using primers Fwd: actgctcgagggaaaacagacccag and Rev: actgaagcttcggcaggtgtagatacgtca, and cloned into the XhoI and HindIII sites of pGL3-Basic (Promega). Subsequently, 5' deletions were generated by PCR using the alternative forward primers in the table below with the reverse primer listed above, and 3' deletion with the alternative reverse primers in the table below and the forward primer listed above. Alternatively, annealed oligo adapters were inserted in the indicated sites to generate the desired mutants.

Table - oligos used for cloning transcriptional reporters

Construct	Direction	Sequence	Method	Cloning sites
TRIM52 full-length reporter	Fwd	actgctcgagggaaaacagacccag	PCR	XhoI-HindIII
TRIM52 full-length reporter	Rev	actgaagcttcggcaggtgtagatacgtca	PCR	XhoI-HindIII
TRIM52 5'-Δ200 mutant	Fwd	agtgctcgagaccctggcccgacgcag	PCR	XhoI-HindIII
TRIM52 5'-Δ370 mutant	Fwd	agtgctcgaggctcacggaaggcagcg	PCR	XhoI-HindIII
TRIM52 5'-Δ600 mutant	Fwd	agtgctcgagcggagagatttatcccccttcc	PCR	XhoI-HindIII
TRIM52 5'-Δ780 mutant	Fwd	agtgctcgagcgagggagctgagaagggg	PCR	XhoI-HindIII
TRIM52 5'-Δ1000 mutant	Fwd	agtgctcgagtgcgtcgcctcgggcag	PCR	XhoI-HindIII
TRIM52 3'-Δ200 mutant	Top	gttaattaagaattca	Oligo adapter	PstI-HindIII
TRIM52 3'-Δ200 mutant	Bot	agcttgaattcttaattaactgca	Oligo adapter	PstI-HindIII
TRIM52 3'-Δ310 mutant	Rev	aagcttggcagaggcgacgcaaaatgacgtc	PCR	BssHII-HindIII
TRIM52 3'-Δ350 mutant	Rev	aagcttggcagctctggagagacgctctagg	PCR	BssHII-HindIII
TRIM52 3'-Δ390 mutant	Rev	aagcttggcaggccgcgcgggccgggag	PCR	BssHII-HindIII

Generation of TRIM52 expression constructs

The full-length *TRIM52* ORF (Genbank accession: NM_032765.3)[2,6] or domain deletions thereof were cloned into the BamHI-PacI sites of a derivative of the lentiviral expression

construct pLX303 containing an SFFV promoter, followed by the coding sequence for the OLLAS tag[7,8]. The C35A point mutant was generated by site-directed mutagenesis PCR (QuickChange XL, Stratagene), and the deletion mutants by truncation or fusion PCR, using the oligonucleotides listed in the table below. For TRIM52 domain fusions, EGFP was cloned into the BamHI–NsiI sites of the pLX-SFFV-OLLAS construct, after which individual TRIM52 domains amplified by PCR (primers in table below) were inserted into the NsiI-PacI sites.

Table - oligos used for cloning TRIM52 mutant expression constructs

Construct	Direction	Sequence	Method	Cloning sites
TRIM52 full-length	Fwd	ctagacgcgtatgctggttatgccactactcccag	PCR	MluI-PacI
TRIM52 full-length	Rev	ctagttaattaattactgattataggccttgctgtgaatgc	PCR	MluI-PacI
TRIM52 C35A	Fwd	cccgtgtccatcagcgctgggcacaacttctg	Site-direct mut PCR	MluI-PacI
TRIM52 C35A	Rev	cagaagttgtgcccagcgctgatggacacggg	Site-direct mut PCR	MluI-PacI
TRIM52-ΔRING	Fwd	ggttacgcgtatcccaacttgcagctagccaac	Truncation PCR	MluI-PacI
TRIM52-ΔBbox	Rev	ggggcaggtgaactgcccctgggtcacacaccctcgg	Fusion PCR	MluI-PacI
TRIM52-ΔBbox	Fwd	gggtgtgtgacccaggggcagttcacctgcccc	Fusion PCR	MluI-PacI
TRIM52-ΔBbox	Rev	cctggtactcctgcaccacctcctcgccctgatcattactccggtttc	Fusion PCR	MluI-PacI
TRIM52-ΔBbox	Fwd	ggagtaatgatcagggcgaggaggtggtgcaggagtaccagg	Fusion PCR	MluI-PacI
TRIM52-ΔCTD	Rev	tttcacttaattaattactggtactcctgcaccacct	Truncation PCR	MluI-PacI

Table - oligos used for cloning EGFP-TRIM52 domain expression constructs

Construct	Direction	Sequence	Method	Cloning sites
EGFP-RING	Fwd	actgatgcatgctggttatgccactactcccag	Truncation PCR	NsiI-PacI
EGFP-RING	Rev	actgttaattaaacgaaagctgcgacgtgtaaag	Truncation PCR	NsiI-PacI
EGFP-RING-Δloop2	Fwd	actgatgcatgctggttatgccactactcccag	Truncation PCR	NsiI-PacI
EGFP-RING-Δloop2	Rev	actgttaattaaacgaaagctgcgacgtgtaaag	Truncation PCR	NsiI-PacI
EGFP-Bbox	Fwd	actgatgcattgctttaaacacaggaagccct	Truncation PCR	NsiI-PacI
EGFP-Bbox	Rev	actgttaattaacaaaggcagcacgctgtg	Truncation PCR	NsiI-PacI
EGFP-CTD	Fwd	actgatgcatgaaataaagttggaaacaactctggtggga	Truncation PCR	NsiI-PacI
EGFP-CTD	Rev	actgttaattaactgattataggccttgctgtgaatg	Truncation PCR	NsiI-PacI

Supplementary Figure legends

Figure S1: TRIM52 is ubiquitously expressed at low levels, yet essential for optimal cell fitness. (a) HEK-293T cells were transfected with the indicated amounts of OLLAS- or MYC-tagged TRIM52 expression plasmids. At 48 h post-transfection whole-cell lysates were harvested and analysed in duplicate; one of the blots was incubated with the A-4 TRIM52 mAb, and the corresponding duplicate blot with either an OLLAS or MYC mAb. Equal exposure times are shown. **(b)** Competition cell fitness assay. Seventy percent EGFP-expressing *TRIM52* knock-down U87MG cells, or CRISPR knock-out RKO cells were mixed with 30% non-fluorescent wild-type cells. Subsequently, their relative abundance in the cell population was measured by flow cytometry at the indicated times, and plotted as a relative fraction of the initial percentage of fluorescent cells. Data represent means and s.d.; U87MG n=2, RKO n=3.

Figure S2. *TRIM52* mRNA is expressed at moderate-low levels in different cell types from a gene-adjacent 93 bp intergenic region. (a) Dual luciferase assays performed with 5'- and 3'- deletion constructs of the *TRIM52* transcriptional reporter in HEK-293T cells. Data represent means and s.d; n=3. **(b)** Publically available ChIP-seq data were mapped to the identified *TRIM52* transcriptional region using the ReMap platform, and the identification frequency of the indicated transcriptional regulators across all different cell lines plotted. Green bars indicate transcription factors targeted in Fig. 2e.

Figure S3. TRIM52 is upregulated during complex cell stress induced by Golgi toxins. (a) U87MG cells were treated/infected with the indicated cytokines/viruses. After 8 and 24 h, mRNA levels for the indicated established response genes were determined by RT-qPCR. **(b-c)** U87MG cells were treated for 8 or 24 h with tunicamycin (Tm), thapsigargin (Tg), or brefeldin A (BFA), after which **(b)** an *XBP1* mRNA splicing assay was performed, or **(c)** mRNA levels were determined by RT-qPCR. **(d)** RKO cells were treated with the Golgi stress inducers BFA or golgicide A (GCA) for 8 or 24 h, after which mRNA levels of the ER stress

marker *HSPA5* were determined by RT-qPCR. RT-qPCR data represent means and s.d.; n=3. ** p=0.01, *** p=0.001.

Figure S4. (a) TRIM52 protein is present at low steady-state levels as a result of rapid proteasomal turn-over. RKO cells expressing Cas9 and either non-targeting or *TRIM52*-targeting sgRNAs were treated with DMSO, MG132 (10 µM), epoxomicin (10 µM), or bortezomib (10 µM) for 4 h, and whole cell lysates analysed for TRIM52 by WB.

Supplementary figures:

Figure S1:

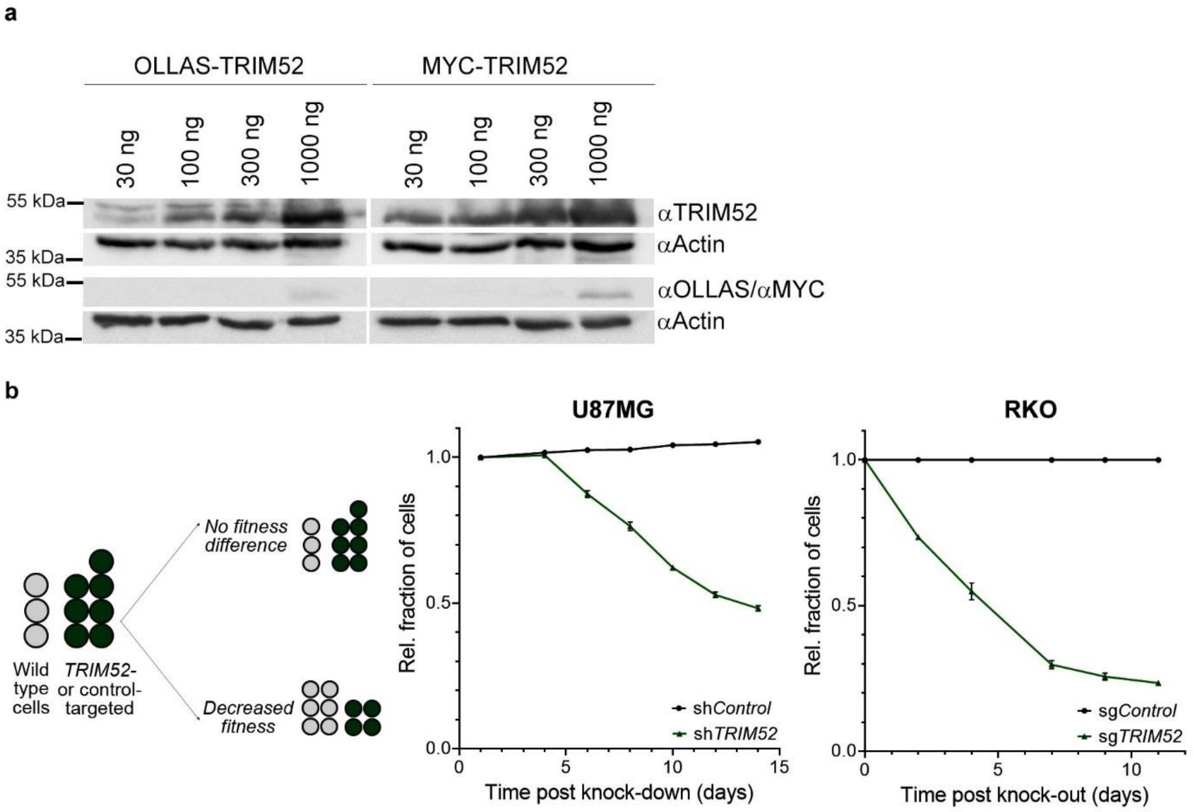

Figure S2:

a

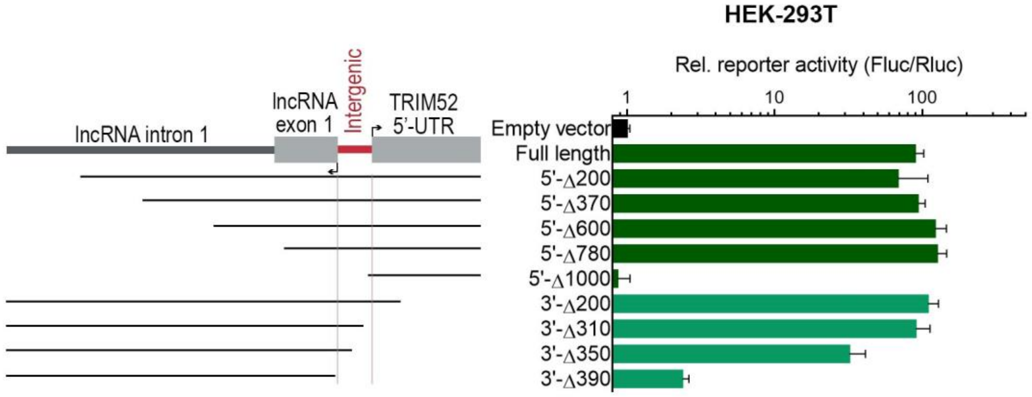
HEK-293T
Rel. reporter activity (Fluc/Rluc)
1
10
100
lncRNA intron 1
lncRNA exon 1
Intergenic
TRIM52 5'-UTR
Empty vector
Full length
5'-Δ200
5'-Δ370
5'-Δ600
5'-Δ780
5'-Δ1000
3'-Δ200
3'-Δ310
3'-Δ350
3'-Δ390

b

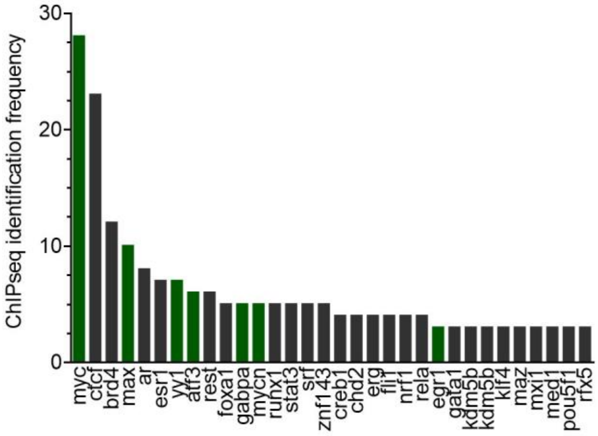
ChIPseq identification frequency
30
20
10
0
myc
ctcf
brd4
max
ar
esr1
yy1
atf3
rest
foxa1
gabpa
mycn
runx1
stat3
srf
znf143
creb1
chd2
erg
fli1
nrf1
rela
egr1
gata1
kdm5b
kdm5b
klf4
maz
mxi1
med1
pou5f1
rfx5

Figure S3:

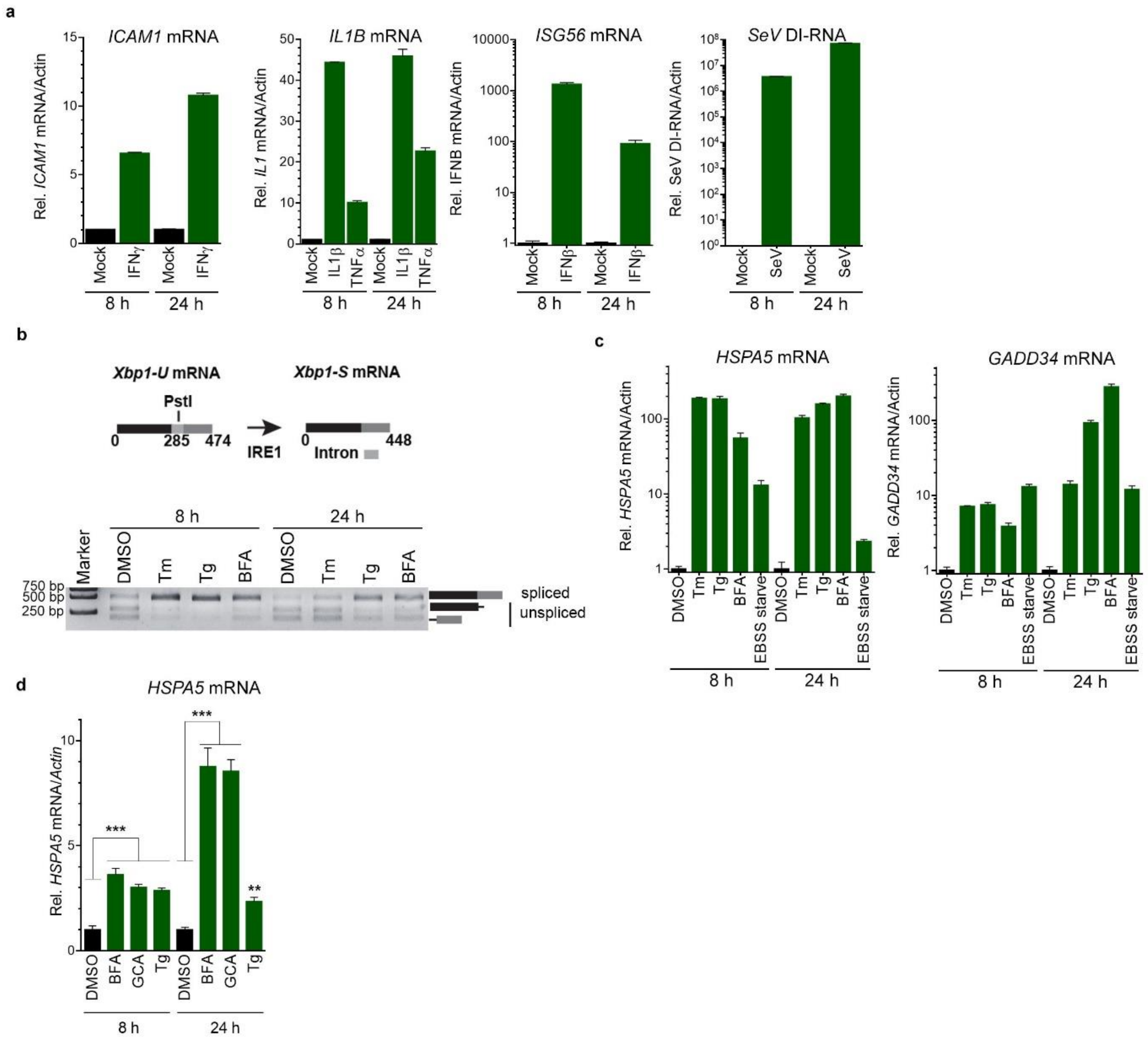

a
ICAM1 mRNA
IL1B mRNA
ISG56 mRNA
SeV DI-RNA
Rel. ICAM1 mRNA/Actin
Rel. IL1 mRNA/Actin
Rel. IFNB mRNA/Actin
Rel. SeV DI-RNA/Actin
Mock
IFNγ
IL1β
TNFα
IFNβ
SeV
8 h
24 h
b
Xbp1-U mRNA
Xbp1-S mRNA
PstI
0
285
474
448
IRE1
Intron
Marker
DMSO
Tm
Tg
BFA
750 bp
500 bp
250 bp
spliced
unspliced
c
HSPA5 mRNA
GADD34 mRNA
Rel. HSPA5 mRNA/Actin
Rel. GADD34 mRNA/Actin
EBSS starve
d
HSPA5 mRNA
Rel. HSPA5 mRNA/Actin
GCA

Figure S4:

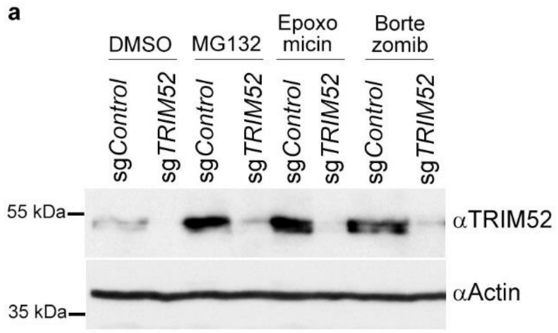
a
DMSO
MG132
Epoxo micin
Borte zomib
sgControl
sgTRIM52
sgControl
sgTRIM52
sgControl
sgTRIM52
sgControl
sgTRIM52
55 kDa
αTRIM52
αActin
35 kDa

Unprocessed blot and gel images

The images displayed below are the unprocessed blot and gel figures used to generate the processed composite figures in the manuscript.

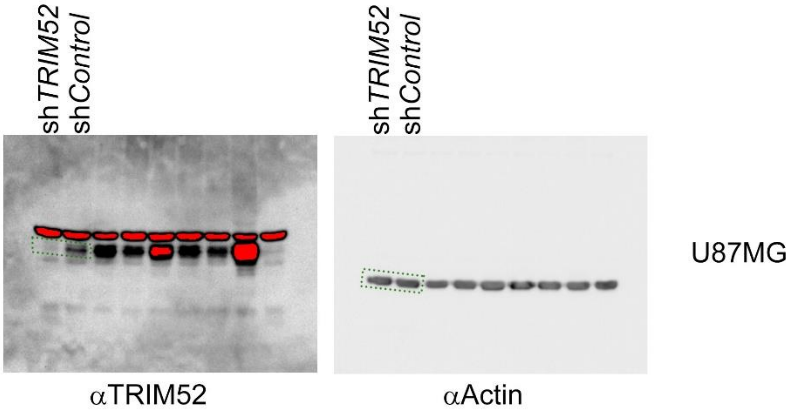

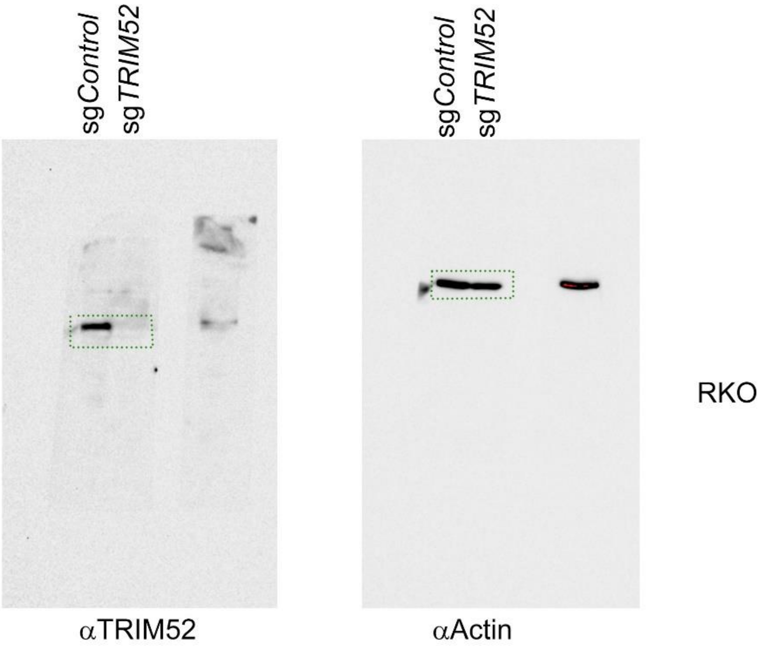

<u>Original blots related to Fig. 1b</u>

Original Western blots of the cropped panels displayed in Fig. 1b. Regions displayed in the main figure are indicated by dotted green lines. The indicated TRIM52 and actin lanes from the U87MG cells are displayed in the main figure as mirror image to match the sample order of the RKO cells. The presented data originate from a longer exposure of the same blot as displayed in Fig. 3d; the negative control is displayed in both images. Both the TRIM52-targeting and non-targeting cells were exposed to DMSO. Only annotated/named lanes are displayed in the main figure. Saturated pixels are indicated in red; exposures with saturated content in relevant areas were not analysed for any of the figures in the manuscript.

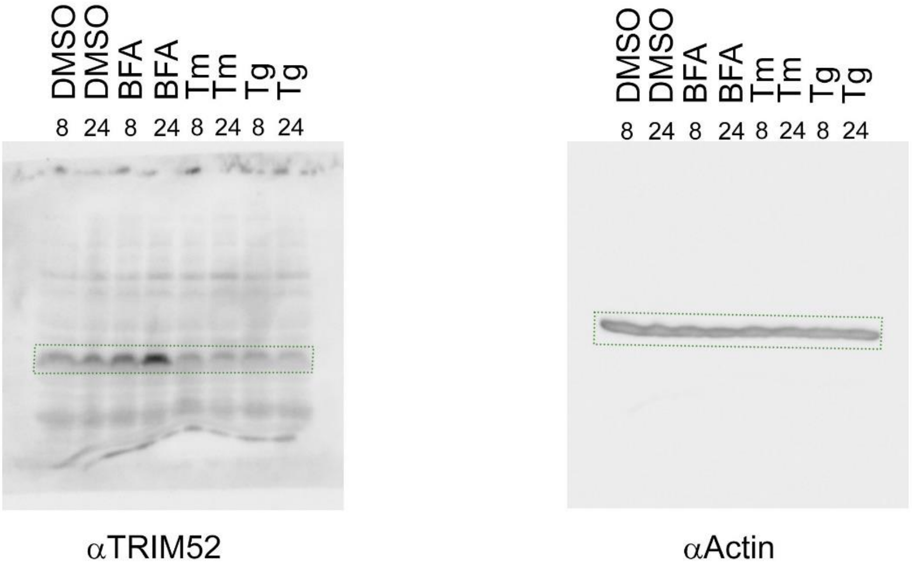

<u>Original blots related to Fig. 3c</u>
Original Western blots of the cropped panels displayed in Fig. 3c. Regions displayed in the main figure are indicated by dotted green lines. Red coloring indicates saturated pixels.

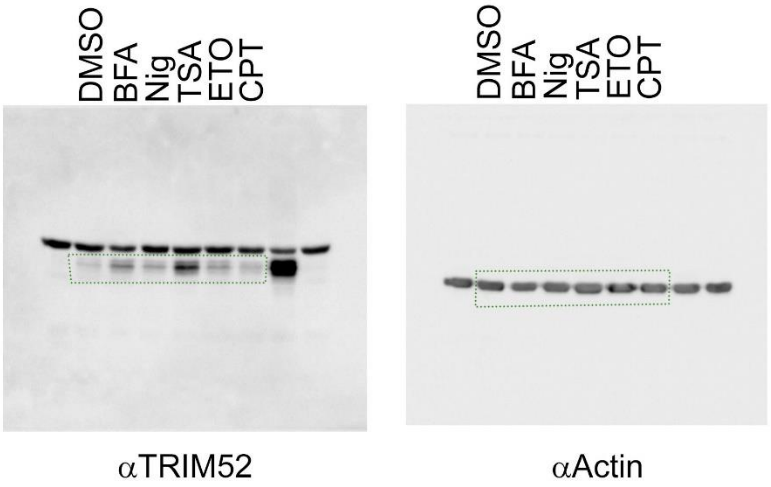

Original blots related to Fig. 3d

Original Western blots of the cropped panels displayed in Fig. 3d. Regions displayed in the main figure are indicated by dotted green lines. The presented data originate from a shorter exposure of the same blot as displayed in Fig. 1c; the negative control is displayed in both images. All annotated samples originate from shControl expressing U87MG cells. Only annotated/named lanes are displayed in the main figure. Red coloring indicates saturated pixels.

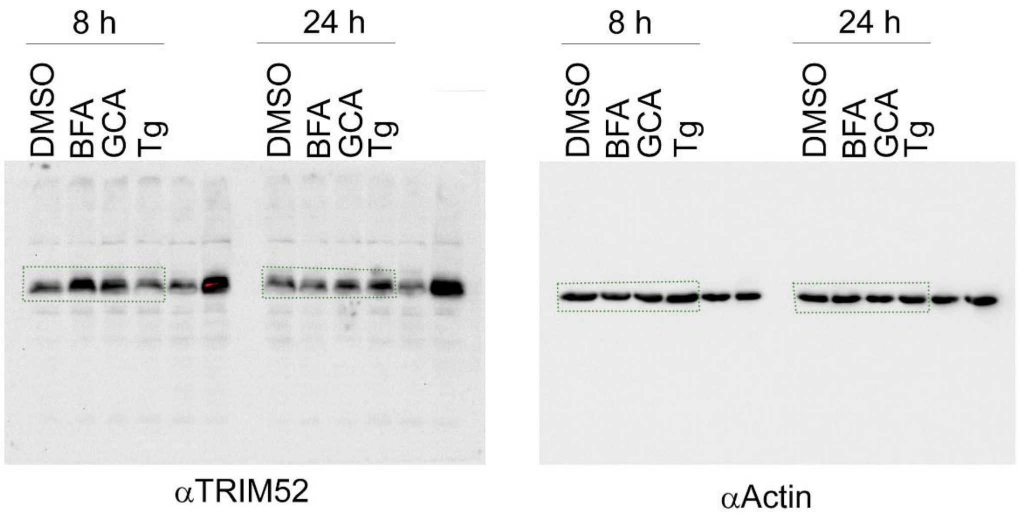

Original blots related to Fig. 3e

Original Western blots of the cropped panels displayed in Fig. 3e. Regions displayed in the main figure are indicated by dotted green lines. Only annotated/named lanes are displayed in the main figure. Red coloring indicates saturated pixels.

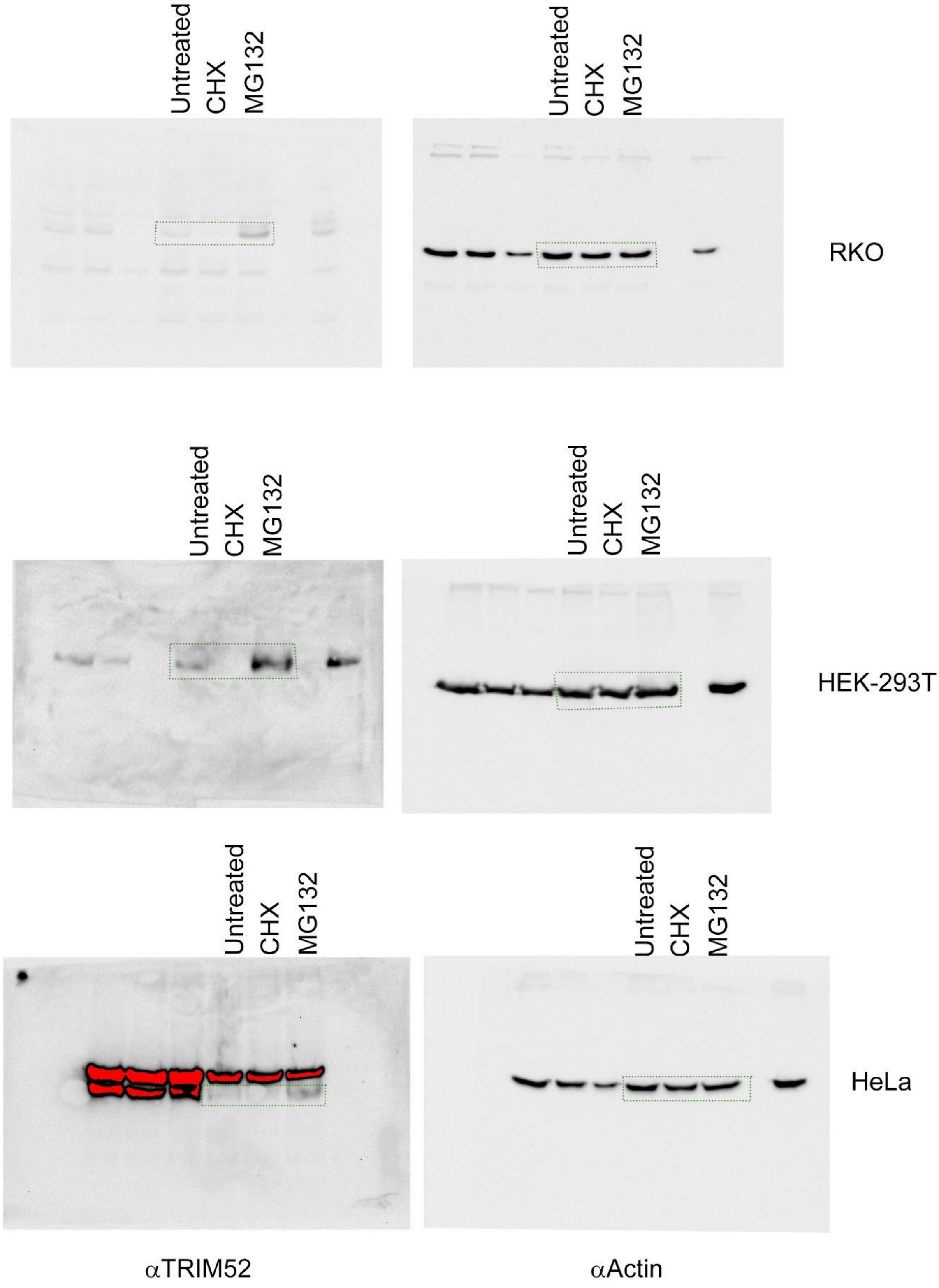

Original blots related to Fig. 4a

Original Western blots of the cropped panels displayed in Fig. 4a. Regions displayed in the main figure are indicated by dotted green lines. Only annotated/named lanes are displayed in the main figure. Red coloring indicates saturated pixels.

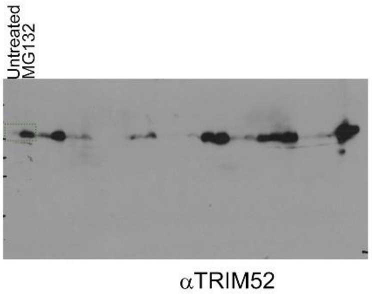

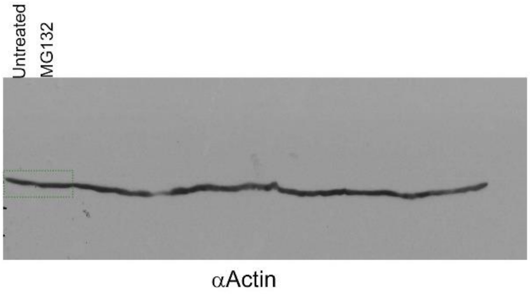

Original blots related to Fig. 4b

Original Western blots of the cropped panels displayed in Fig. 4b. Regions displayed in the main figure are indicated by dotted green lines. Only annotated/named lanes are displayed in the main figure. Red coloring indicates saturated pixels.

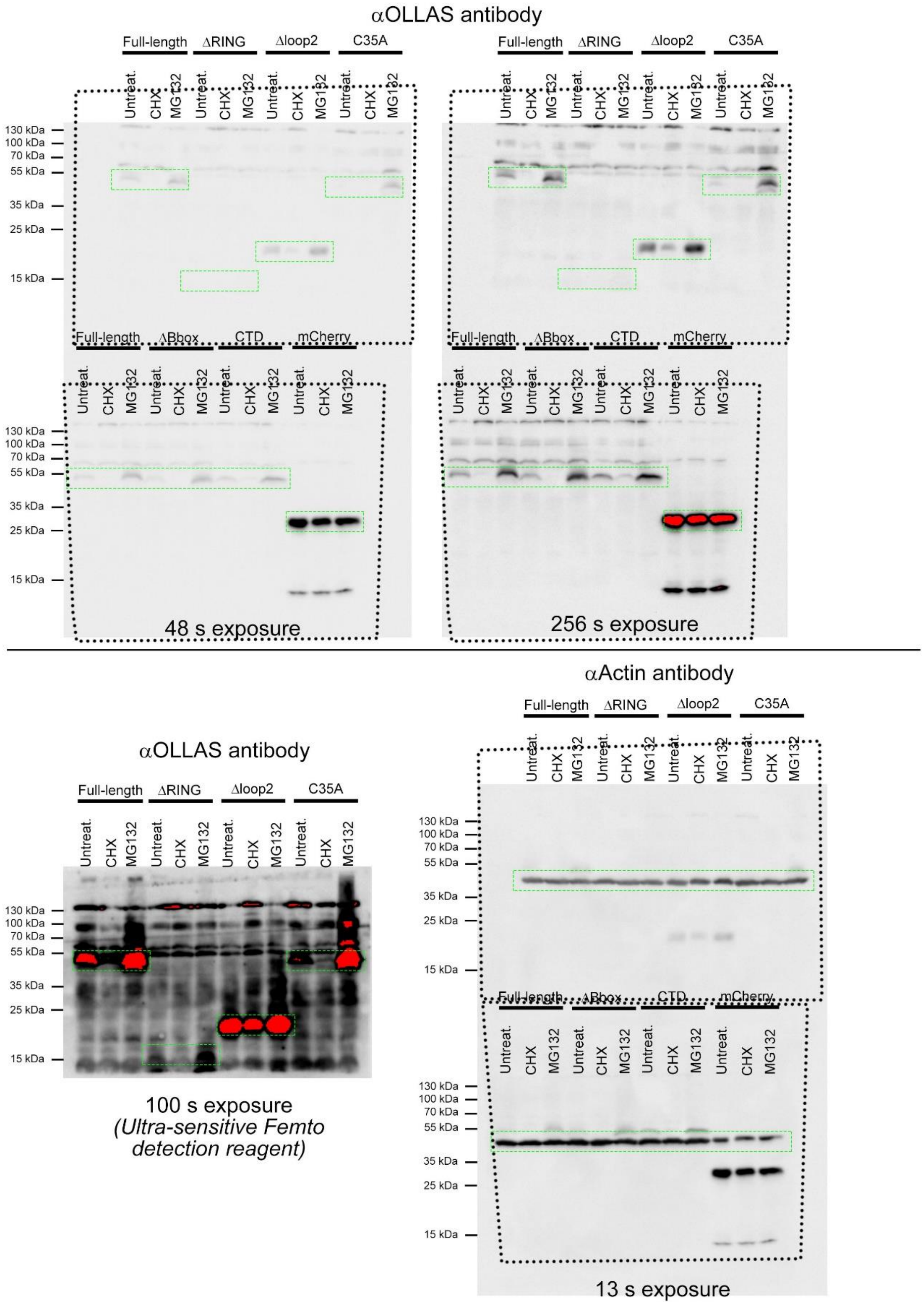

Original blots related to Fig. 4d

Original Western blots of the cropped panels displayed in Fig. 4d. For figure panels several exposure times of the same blots are shown. Red indicates saturated exposure as determined by the Chemidoc machine; only non-saturated parts were used to generate the corresponding compound figure in the main text. Bands of interest represented in the corresponding main figure are indicated in the dotted green rectangles. The outlines of each blot are indicated with black dotted lines. Blots were first analysed with αOLLAS antibody, and subsequently with αActin without stripping in between; some of the original OLLAS signal is visible in the actin blot.

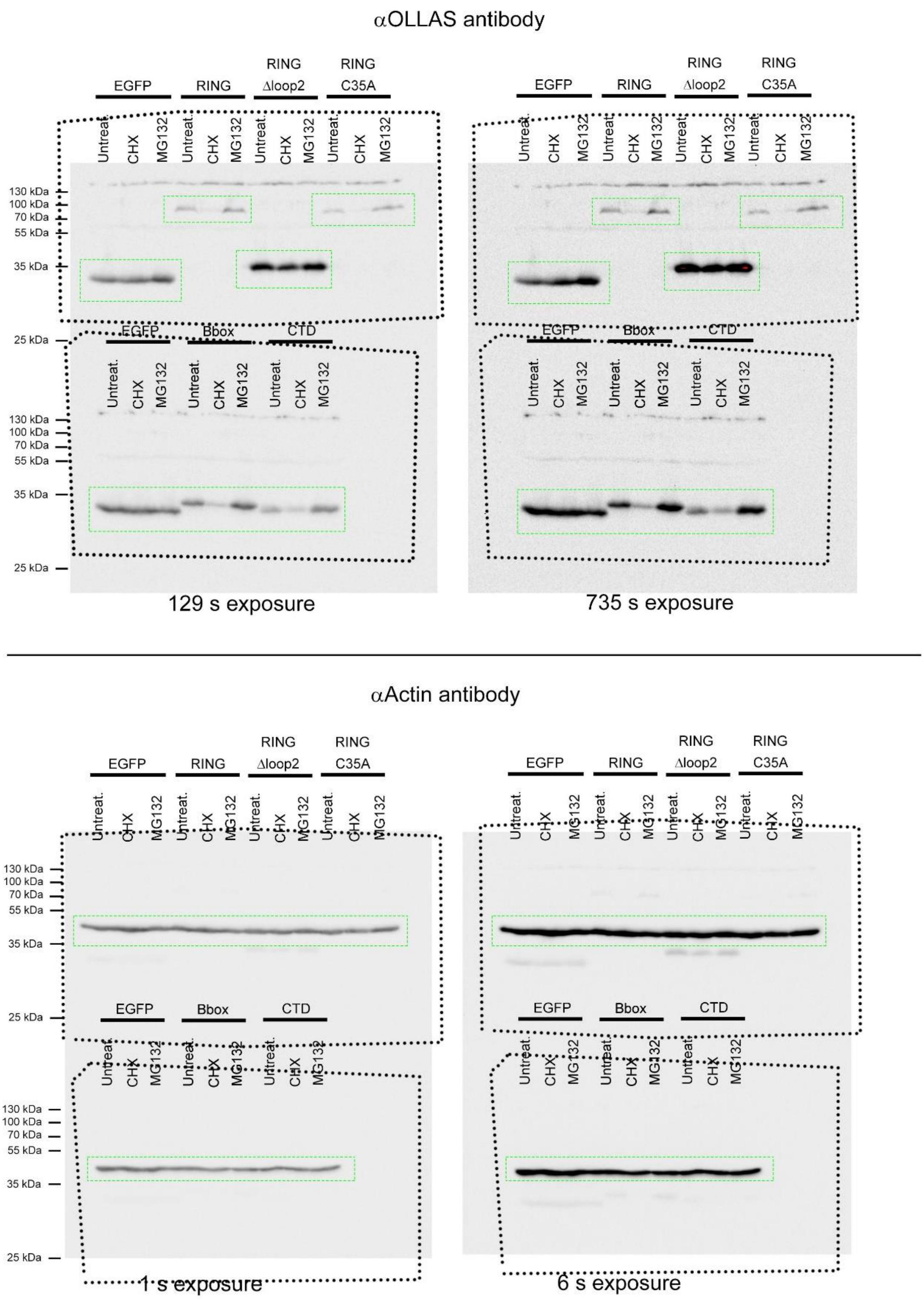

Original blots related to Fig. 4e

Original Western blots of the cropped panels displayed in Fig. 4d. For figure panels several exposure times of the same blots are shown. Red indicates saturated exposure as determined by the Chemidoc machine; only non-saturated parts were used to generate the corresponding compound figure in the main text. Bands of interest represented in the corresponding main figure are indicated in the dotted green rectangles. The outlines of each blot are indicated with black dotted lines. Blots were first analysed with αOLLAS antibody, and subsequently with αActin without stripping in between; some of the original OLLAS signal is visible in the actin blot.

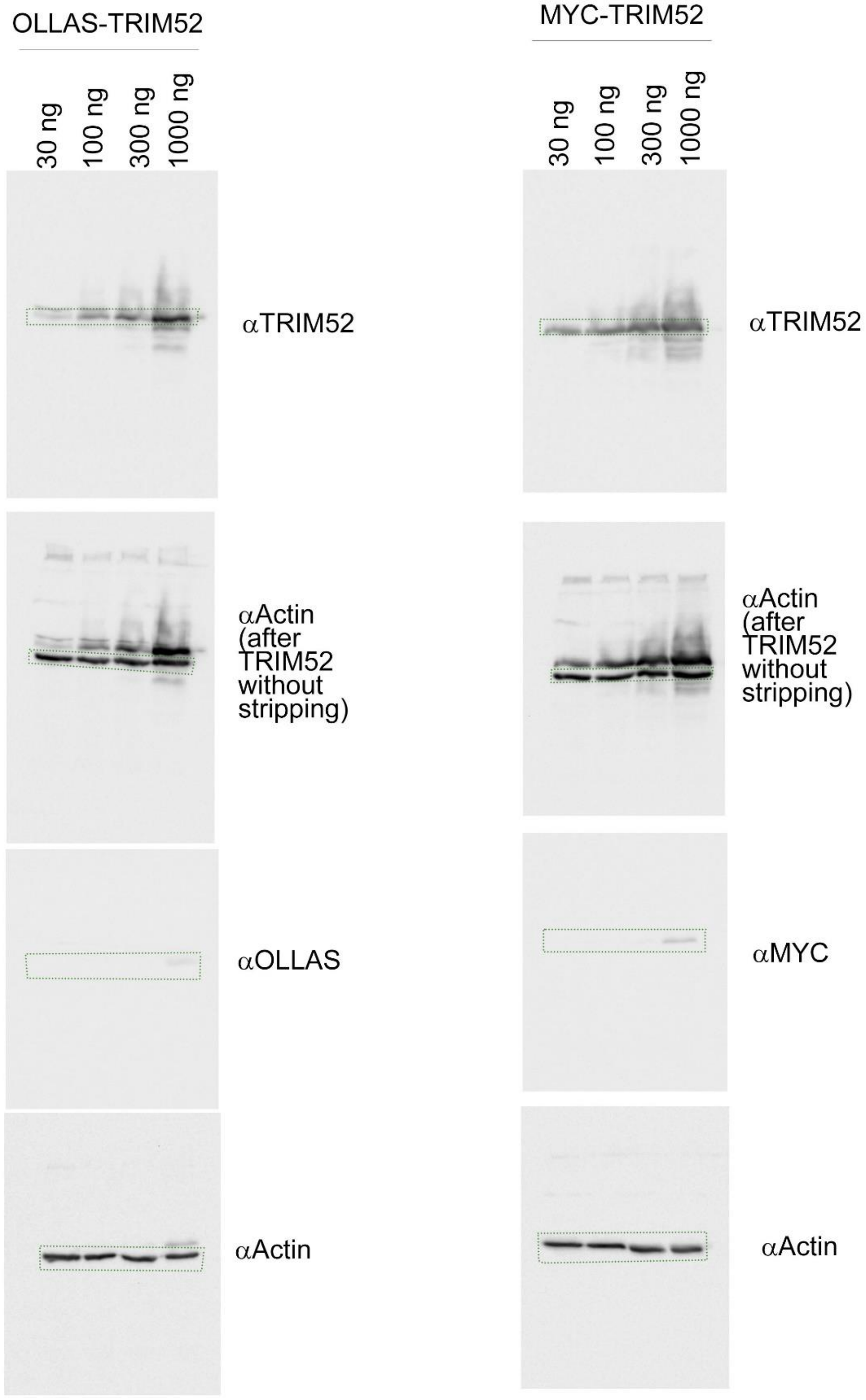

<u>Original blots related to Fig. S1</u>

Original Western blots of the cropped panels displayed in Fig. S1. Blots were first analysed with αTRIM52 antibody, and subsequently with αActin without stripping in between; some of the original TRIM52 signal is visible in the Actin blot. Regions displayed in the main figure are indicated by dotted green lines. Only annotated/named lanes are displayed in the main figure. Red coloring indicates saturated pixels.

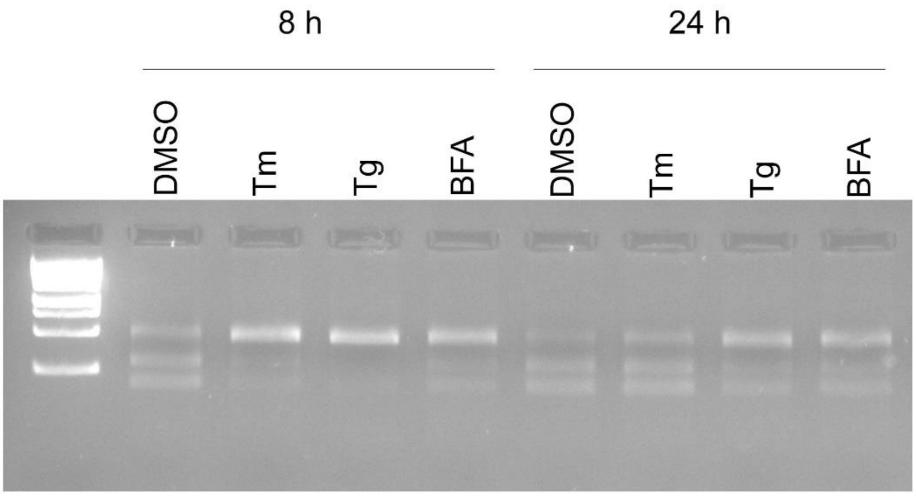

Original agarose gel related to Fig. S3b

Original agarose gel of the cropped panels displayed in Fig. S3b. Black/white values were inverted equally across the complete image to generate the main figure.

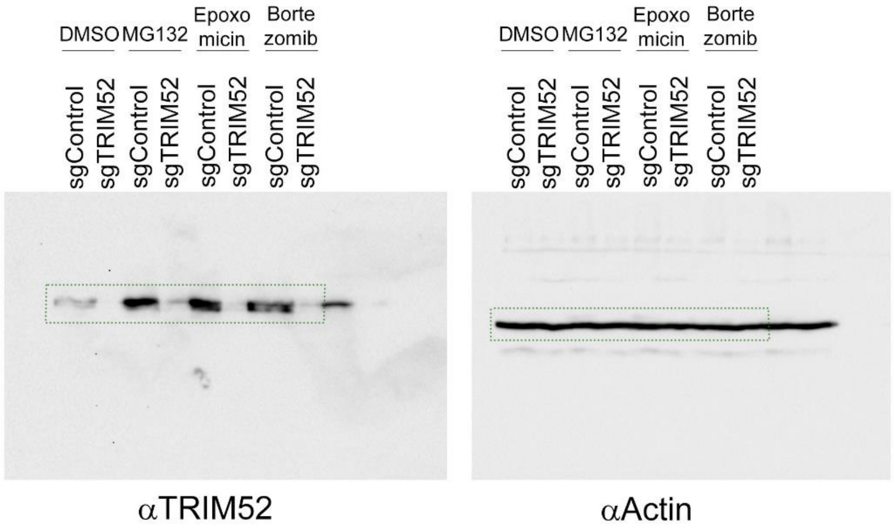

Original blots related to Fig. S4a

Original Western blots of the cropped panels displayed in Fig. S4a. Regions displayed in the main figure are indicated by dotted green lines. Only annotated/named lanes are displayed in the main figure. Red coloring indicates saturated pixels.

References:

1. Ovcharenko, I., Nobrega, M. A., Loots, G. G. & Stubbs, L. ECR Browser: a tool for visualizing and accessing data from comparisons of multiple vertebrate genomes. *Nucleic Acids Res.* **32**, W280-286 (2004).
2. Fellmann, C. *et al.* An optimized microRNA backbone for effective single-copy RNAi. *Cell Rep.* **5**, 1704–1713 (2013).
3. Benke, S. *et al.* Human tripartite motif protein 52 is required for cell context-dependent proliferation. *Oncotarget* **9**, 13565–13581 (2018).
4. Sanjana, N. E., Shalem, O. & Zhang, F. Improved vectors and genome-wide libraries for CRISPR screening. *Nat. Methods* **11**, 783–784 (2014).
5. Wang, T., Wei, J. J., Sabatini, D. M. & Lander, E. S. Genetic screens in human cells using the CRISPR-Cas9 system. *Science* **343**, 80–84 (2014).
6. Versteeg, G. A. *et al.* The E3-ligase TRIM family of proteins regulates signaling pathways triggered by innate immune pattern-recognition receptors. *Immunity* **38**, 384–398 (2013).
7. Park, S. H. *et al.* Generation and application of new rat monoclonal antibodies against synthetic FLAG and OLLAS tags for improved immunodetection. *J. Immunol. Methods* **331**, 27–38 (2008).
8. Yang, X. *et al.* A public genome-scale lentiviral expression library of human ORFs. *Nat. Methods* **8**, 659–661 (2011).
